# A Vesicular Stomatitis Virus Replicon-Based Bioassay for the Rapid and Sensitive Determination of Multi-Species Type I Interferon

**DOI:** 10.1371/journal.pone.0025858

**Published:** 2011-10-05

**Authors:** Marianne Berger Rentsch, Gert Zimmer

**Affiliations:** Institut für Viruskrankheiten und Immunprophylaxe, Mittelhäusern, Switzerland; McMaster University, Canada

## Abstract

Type I interferons (IFN) comprise a family of cytokines that signal through a common cellular receptor to induce a plethora of genes with antiviral and other activities. Recombinant IFNs are used for the treatment of hepatitis C virus infection, multiple sclerosis, and certain malignancies. The capability of type I IFN to suppress virus replication and resultant cytopathic effects is frequently used to measure their bioactivity. However, these assays are time-consuming and require appropriate biosafety containment. In this study, an improved IFN assay is presented which is based on a recombinant vesicular stomatitis virus (VSV) replicon encoding two reporter proteins, firefly luciferase and green fluorescent protein. The vector lacks the essential envelope glycoprotein (G) gene of VSV and is propagated on a G protein-expressing transgenic cell line. Several mammalian and avian cells turned out to be susceptible to infection with the complemented replicon particles. Infected cells readily expressed the reporter proteins at high levels five hours post infection. When human fibroblasts were treated with serial dilutions of human IFN-β prior to infection, reporter expression was accordingly suppressed. This method was more sensitive and faster than a classical IFN bioassay based on VSV cytopathic effects. In addition, the antiviral activity of human IFN-λ (interleukin-29), a type III IFN, was determined on Calu-3 cells. Both IFN-β and IFN-λ were acid-stable, but only IFN-β was resistant to alkaline treatment. The antiviral activities of canine, porcine, and avian type I IFN were analysed with cell lines derived from the corresponding species. This safe bioassay will be useful for the rapid and sensitive quantification of multi-species type I IFN and potentially other antiviral cytokines.

## Introduction

IFN-α and IFN-β are structurally related cytokines of the type I interferon family which mediate an early innate immune response to viral infections. There are 13 distinct IFN-α genes present in the human genome, and a single gene encoding for IFN-β. These genes are transcriptionally activated in cells sensing a virus infection through pattern recognition receptors such as the retinoic acid inducible gene I (RIG-I) helicase. Following secretion, IFN-α and IFN-β act similarly by binding to a common, ubiquitously expressed IFN-α/β receptor resulting in the activation of the JAK/STAT signal transduction pathway and transcription of “IFN-induced genes” [1], [2]. Several of these genes encode for proteins with strong antiviral activity, i.e. Mx protein, protein kinase R, and 2′-5′oligo(A) synthetase [3]. Due to their autocrine action, type I IFN may attenuate virus replication in infected cells. Probably more important is the paracrine action of type I IFN, which induces an antiviral state in previously uninfected cells, thereby blocking virus dissemination in the organism. In addition to this “classical” antiviral function, type I IFNs are known to affect cell proliferation and differentiation, to modulate the immune response, to inhibit angiogenesis, and to promote apoptosis [4], [5]. Genetically engineered type I IFNs are currently in clinical use for the treatment of multiple sclerosis [6], chronic hepatitis C virus infection [7], and certain types of cancer [8], [9]. An issue of increasing importance is the determination of neutralizing antibodies that are induced in some patients following recombinant IFN therapy [10].

Apart from IFN-α/β, cytokines such as IFN-γ (type II IFN) and IFN-λ (type III IFN) exhibit antiviral activities, although they bind to distinct receptors. In particular, type III IFNs induce transcriptional activation of antiviral genes similar to those activated by type I IFN. Type III IFNs act primarily on epithelial cells [11] and probably play an important role in the innate immune response of epithelial tissues to virus infections [12], [13].

The accurate determination of antiviral IFN activity is a cumbersome issue. In the “classical” bioassay, serial dilutions of both a test sample with unknown IFN activity and a type I IFN standard are incubated with an appropriate cell line prior to infection with a cytolytic virus such as vesicular stomatitis virus (VSV), encephalomyocarditis virus, or Sendai virus [14], [15]. The reciprocal value of the highest type I IFN dilution mediating protection of 50% of the cells from virus-induced cytopathic effects (CPE) is defined as one unit of type I IFN per volume. This classical IFN bioassay is time-consuming because the CPE normally needs 24 hours or more to develop. A faster readout can be achieved with recombinant viruses expressing reporter proteins such as green fluorescent protein (GFP) or firefly luciferase [16], [17], [18], [19]. However, any work with live virus requires appropriate biosafety containment. For example, a recently published IFN bioassay can only be performed in biosafety level 3 (BSL-3) facilities, because the test makes use of a recombinant Rift Valley Fever virus [19]. Viral replicon-based bioassays that take advantage of disabled propagation-incompetent viruses may provide an attractive alternative to live virus-based bioassays. A human hepatoma cell line harbouring a selectable hepatitis C virus replicon has been successfully employed for the measurement of type I IFN from patients with chronic hepatitis C virus infection [20]. However, as type I IFNs act in a species-dependent manner, this system may not be applicable to animal IFNs. Transgenic cell lines expressing a reporter gene under control of an IFN-responsive promoter may also be used to determine IFN activity under biosafe conditions [21], [22]. However, it is difficult to simply relate transcriptional reporter gene activation to antiviral activity.

In this report, a novel type I IFN bioassay is presented, which is based on BSL-1-classified VSV replicon particles. The assay is highly sensitive and quantifiable due to the expression of a firefly luciferase reporter gene. In addition, the assay can be rapidly performed within 6 to 7 hours and may be used to determine the antiviral activity of IFNs from humans as well as other species. Thus, this bioassay may be of general interest for all those who want to determine the antiviral activity of cytokines such as type I IFNs.

## Materials and Methods

### Cells

BHK-21 cells were obtained from the German Cell Culture Collection (DSZM, Braunschweig, Germany) and grown in Earle's minimal essential medium (EMEM) supplemented with 5% fetal bovine serum (FBS). BHK-G43, a transgenic BHK-21 cell clone expressing VSV G protein in a regulated manner, was maintained as described previously [23]. The porcine kidney cell line PK-15 (ATCC, Manassas, VA) was propagated in Dulbecco's modified Eagle medium supplemented with nonessential amino acids, 1 mM Na-pyruvate and 5% horse serum. D-17 canine osteosarcoma cells (ATCC), Calu-3 human lung adenocarcinoma cells (ATCC), and NHDF normal human dermal fibroblasts (Lonza, Cologne, Germany) were maintained in EMEM with 10% FBS. The UMNSAH/DF-1 (DF-1) chicken fibroblast cell line (ATCC) was maintained in Dulbecco's Modified Eagle's Medium and 10% FBS. All cell lines were cultured at 37°C in a humidified atmosphere containing 5% CO_2_, except DF-1 cells which were kept at 39°C. BALB 3T3 fibroblasts (subclone A31) were kindly provided by N. Pringle, University College, London, UK, and maintained in Dulbecco's Modified Eagle's Medium.

### Interferons

Human and murine IFN-β and human IFN-λ (IL-29) were purchased from PBL InterferonSource (Piscataway, NJ). Recombinant porcine IFN-α1 [24] was kindly provided by Nicolas Ruggli (IVI, Mittelhäusern, Switzerland). Recombinant chicken IFN-α [25] was kindly provided by Peter Stäheli (University of Freiburg, Germany). Canine IFN-β [26] was kindly provided by Philippe Plattet (Department of Clinical Research and Veterinary Public Health, University of Bern).

### Generation of VSV*ΔG(Luc) replicon particles

**Figure 1:**
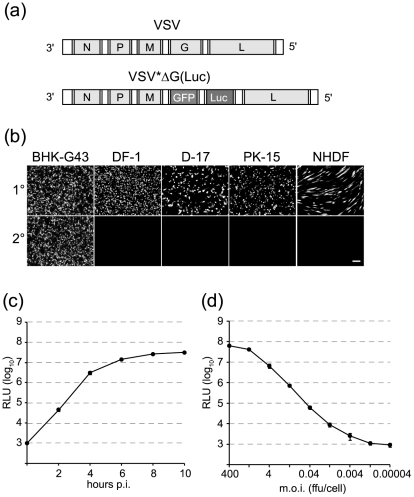
Expression of firefly luciferase and GFP reporter genes by a propagation-incompetent VSVΔG replicon. (a) Genome maps of authentic VSV and recombinant VSV*ΔG(Luc) vector. VSV*ΔG(Luc) lacks the glycoprotein (G) gene and drives expression of both enhanced green fluorescent protein (GFP) and firefly luciferase (Luc). (b) Cell lines derived from different species were infected with 4 ffu/cell of VSV*ΔG(Luc) (1^st^ cycle). Sixteen hours post infection, the undiluted cell culture supernatant was passed to fresh cells (2^nd^ cycle). GFP expression in live cells was monitored 6 h post infection using an inverse fluorescence microscope. Bar, 100 µm. (c) Firefly luciferase activity in BHK-21 cell lysates at different times post infection with 4 ffu/cell of VSV*ΔG(Luc). (d) Firefly luciferase levels in BHK-21 cell lysates 5 h post infection by VSV*ΔG(Luc) with the indicated multiplicities of infection (m.o.i.).

A plasmid-based rescue system [27] was used to generate a G-deleted VSV driving expression of firefly luciferase and eGFP. The previously described genomic plasmid pVSV*ΔG(HA) containing 6 distinct transcription units (N-P-M-HA-eGFP-L) [28] was modified by replacing the influenza virus HA gene in the fourth position with eGFP taking advantage of *Mlu*I and *Bst*EII endonuclease restriction sites upstream and downstream of the HA ORF, respectively. The firefly luciferase gene was amplified from the pBI-L plasmid (Clontech) by *Pfu* PCR and inserted into the fifth transcrition unit using *Xho*I and *Nhe*I endonuclease restriction sites. The resulting plasmid was designated pVSV*ΔG(Luc) and contained 6 genes in the order N-P-M-eGFP-Luc-L (Fig. 1).

For generation of VSV*ΔG(Luc) replicon particles, BHK-G43 helper cells were grown in 100-mm diameter culture dishes to 90% confluence and infected with recombinant modified vaccinia virus Ankara (5 pfu per cell) expressing T7 RNA polymerase (MVA-T7, a gift of Gerd Sutter, München, Germany). MVA-T7 has been classified by the German Central Committee for Biosafety as a BSL-1 organism (reference number 6790-10-14). Ninety minutes post infection, the medium was replaced with fresh EMEM containing 5% FBS and 10^−9^ M mifepristone (Sigma-Aldrich, Buchs, Switzerland) to induce VSV G expression [23]. Subsequently, the cells were transfected with 10 µg of pVSV*ΔG(Luc), 3 µg of pTM1-N, 5 µg of pTM1-P, and 2 µg of pTM1-L [28] using Lipofectamine™ 2000 transfection reagent (Invitrogen, Basel, Switzerland). The cells were trypsinized 24 hours post transfection and seeded into T75 flasks along with an equal number of fresh BHK-G43 cells. The cells were further incubated at 37°C for 24 hours in the presence of 10^−9^ M mifepristone. The cell culture supernatant was clarified by low-speed centrifugation and passed through a 0.20-µm-pore size filter to deplete vaccinia virus. VSV*ΔG(Luc) was further propagated on mifepristone-induced BHK-G43 cells and stored frozen at −70°C. To determine infectious virus titers, confluent BHK-21 grown in 96-well microtiter plates were inoculated in duplicate with 40 µl of serial tenfold virus dilutions for 1 h at 37°C. The wells additionally received 60 µl of EMEM and were incubated for 20 h at 37°C. The infectious titers were calculated according to the number of GFP-expressing cells/well and expressed as fluorescence-forming units per milliliter (ffu/ml).

MVA-T7 was titrated on DF-1 cells grown in 96-well microtiter plates. The cells were inoculated with tenfold serial virus dilutions for 90 min and overlayed with medium containing 0.9% methylcellulose. Following incubation for 48 h at 39°C, the cells were fixed with 3% paraformaldehyde, permeabilized with 0.25% Triton X-100, and subsequently incubated with the TW2.3 monoclonal antibody directed to the vaccinia E3L protein (kindly provided by Jonathan Yewdell, NIH, Bethesda, USA) and anti-mouse IgG conjugated to horseradish peroxidase (DAKO). Infected cell foci were visualized with the AEC peroxidase substrate and expressed as plaque-forming units per milliliter (pfu/ml). Using this assay, the final VSV replicon particle preparations proved to be free of MVA-T7.

### Bioassays for determining antiviral activity

Serial twofold or fourfold dilutions of type I IFN were prepared with cell culture medium containing 5% FBS. The IFN dilutions (100 µl) were added in quadruplicates to confluent cells grown in 96-wells (5×10^4^ cells/well) and incubated for either 1, 2, 4, or 20 hours at 39°C (DF-1 cells) or 37°C (all other cell lines). The cells were infected with VSV*ΔG(Luc) (m.o.i. of 5) and incubated for 5 hours at 37°C. The medium was aspirated and 30 µl of luciferase lysis buffer (Biotium Inc., Hayward, CA) was added to the cells. The cell lysates were stored at −20°C. Firefly luciferase activity was determined with a Centro LB 960 luminometer (Berthold Technologies). Luminescence was recorded for 1 s following injection of 30 µl of D-luciferin substrate (Biotium) to white 96-well plates containing 6-µl aliquots of cell lysate. The relative antiviral activity was calculated according to the following formula: Antiviral Activity (%) = 100 - [(RLU_+IFN_ – Blank)×100/(RLU_−IFN_ - Blank)]. Mock-infected cell lysates served as blanks and relative light units (RLU) detected with these samples were subtracted from the readings taken from VSV*ΔG(Luc)-infected cell lysates. RLU_+IFN_ represents the RLU values from IFN-treated cells and RLU_−IFN_ the readings taken from reference cells, which had not received any type I IFN.

A conventional type I IFN bioassay was performed by incubating 96-well cell cultures for 20 hours with twofold dilutions of type I IFN as described above. The cells were infected with propagation-competent VSV (m.o.i. of 1 pfu/cell) and incubated until a cytopathic effect (CPE) was evident in mock-treated control cells. The cells were washed twice with PBS and stained for 1 h with 0.1% crystal violet in 10% formalin. The plates were washed with tap water to remove excess crystal violet and dried. The dye was dissolved by adding 100 µl of 70% ethanol to each well. The absorbance of crystal violet at 595 nm was determined with a microplate reader. The IFN titer was calculated as the reciprocal of the last IFN dilution causing 50% inhibition of virus-induced CPE (50 % reduction of absorbance at 595 nm compared to mock-infected cells) and was expressed as IFN units per volume. Alternatively, antiviral activity was calculated according to the following formula: Antiviral activity (%) = (OD595_+IFN_ – OD595_−IFN_)×100/(OD595_Mock_ – OD595_−IFN_). OD595_+IFN_ and OD595_−IFN_ represent the absorbance of IFN-treated and non-treated cells following infection with VSV and staining with crystal violet, respectively. OD595_Mock_ denotes the absorbance of non-infected cells.

### Statistical analysis

Mean values and standard deviation were calculated. Statistical analysis was performed using the paired Student's t-test. P<0.05 was considered significant.

## Results

### Generation of the VSV*ΔG(Luc) replicon

Previously, a VSV replicon vector was generated by replacing the glycoprotein G gene of VSV with the hemagglutinin (HA) gene of an H7N1 influenza virus and inserting an extra transcription unit into the HA-L intergenic junction to drive expression of a modified GFP gene [28]. In the present work, this vector was modified by inserting the firefly luciferase gene into the transcription unit at position 5 (thereby replacing the GFP gene) and exchanging the HA gene with the GFP gene (Fig. 1a). The resulting vector, VSV*ΔG(Luc), was propagated on BHK-G43 helper cells which express the VSV G protein in an inducible manner [23]. Up to 10^9^ virus replicon particles (VRP) were released into the cell culture supernatant 24 h post infection (data not shown). Several mammalian and avian cells were found to be susceptible to infection with the VRPs in line with previous observations on the very broad cell tropism of VSV [29]. Infected cells were readily detected (5 to 6 h post infection) by means of GFP reporter expression (Fig. 1b). However, non-helper cells were unable to complement the VSV G deletion and thus could not produce infectious progeny virus. Importantly, VSV*ΔG(Luc) did not induce type I IFN in the cell lines analysed (data not shown). This can be ascribed to the host shut-off activity of the VSV matrix protein [30].

Infection of BHK-21 cells with VSV*ΔG(Luc) using a multiplicity of infection (m.o.i.) of 4 ffu/cell resulted in increasing firefly luciferase reporter activity with time (Fig. 1c). In all subsequent experiments, luciferase activity was generally recorded at 5 h post infection as the signal-to-noise ratio was sufficiently high at this time. When BHK-21 cells were infected with different virus dose rates, luciferase reporter activity increased linearly between 0.004 and 40 ffu/cell (Fig. 1d). Although 95% of the cells showed GFP fluorescence following infection with an m.o.i. of 4 ffu/cell (not shown), an m.o.i. of 40 ffu/cell caused an even tenfold higher luciferase activity suggesting that multiple infections add to the overall replication/transcription rate of the virus genome in a single cell. In all subsequent experiments an m.o.i. of 5 ffu/cell was generally used, as this guaranteed a signal-to noise ratio of about 4 log_10_.

### Quantification of type I IFN antiviral activity

**Figure 2:**
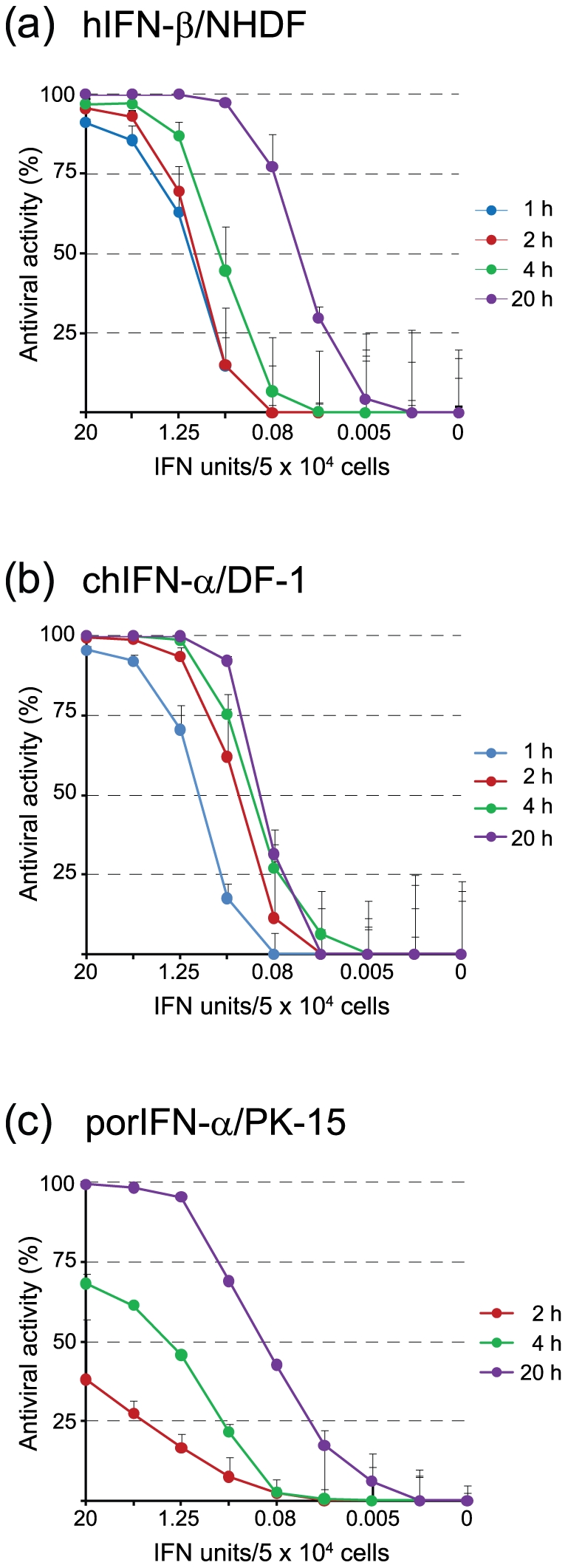
Determination of mammalian and avian type I IFN bioactivity. (a) NHDF (human fibroblasts), (b) DF-1 (chicken fibroblasts), and (c) PK-15 (porcine kidney) cells in 96-well plates were incubated for the indicated times with fourfold serial dilutions of type I IFN from the homologous species and subsequently infected with VSV*ΔG(Luc) (5 ffu/cell). Five hours post infection, the cells were lysed and firefly luciferase activity was recorded. Reference cells did not receive any type I IFN and demonstrated no antiviral activity. The axes of abscissas indicate the activities of the type I IFNs as determined with a conventional CPE-based IFN bioassay.

The luciferase reporter activity in VSV*ΔG(Luc)-infected cells depends on the replication/transcription levels of the RNA replicon. Thus, a reduction in viral genome replication as a consequence of type IFN action should lead to correspondingly lower luciferase levels. To test this hypothesis, normal human dermal fibroblasts (NHDF) were incubated for various time periods with serial dilutions of human IFN-β before infection with VSV*ΔG(Luc). A dose-dependent effect of IFN-β on firefly luciferase reporter activity was observed after 1 hour of incubation (Fig. 2a). About 1 unit of IFN-β led to 50% suppression of luciferase activity. Extending the incubation time to 2 hours did not further improve the sensitivity of the test (p>0.05 for the 0.08 to 1.25 IFN units range). However, if the cells were treated for 20 hours, the dose response curve was significantly shifted to lower IFN units (p≤0.001 for the 0.02 to 20 units range). Approximately 0.05 units of IFN-β were now sufficient to suppress reporter activity by 50%. These results indicate that an IFNβ-induced antiviral state in NHDF cells can be detected as early as 1 hour after addition of IFN-β, although the sensitivity of the assay is higher if the cells were incubated with IFN-β for prolonged time.

VSV*ΔG(Luc) was also used to quantify the activities of porcine and chicken IFN-α. While the dose response curve of chicken IFN-α on DF-1 chicken fibroblasts (Fig. 2b) was similar to the one of human IFN-β on NHDF (Fig. 2a), porcine IFN-α showed a different kinetics on PK-15 porcine kidney cells. In these cells, a full antiviral state was accomplished only after 20 hours of treatment indicating that PK-15 cells respond rather slowly to the action of type I IFN (Fig. 2c). Thus, the assay is applicable to type I IFN from different species but may perform differentially on distinct cell types.

**Figure 3:**
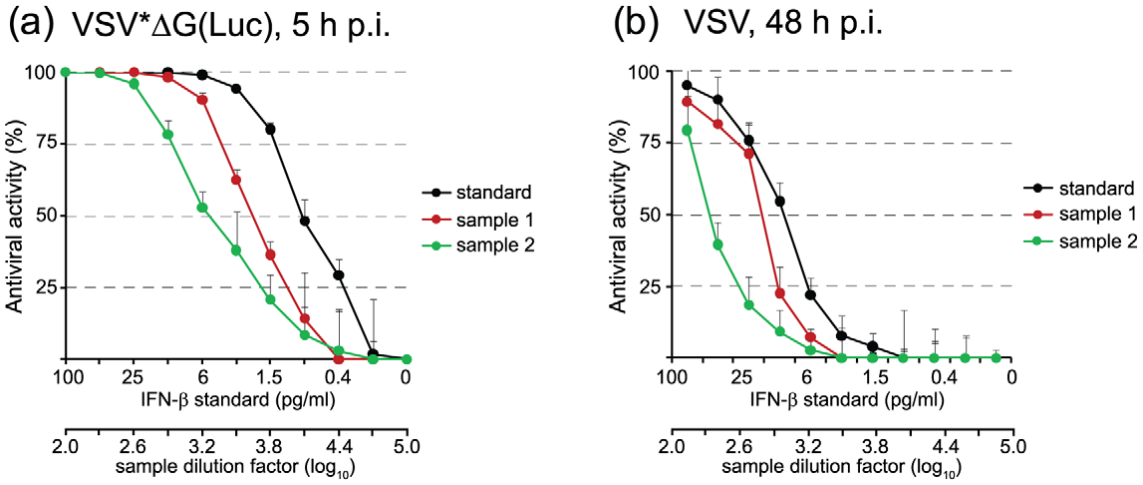
Validation of the VSV*ΔG(Luc) replicon bioassay. Serial twofold dilutions of IFN-β standard (starting with 100 pg/ml) and of two different test samples containing type IFN of unknown activity (starting with dilution 1/100) were prepared and incubated with NHDF for 20 h at 37°C. (a) Five hours post infection with VSV*ΔG(Luc) (m.o.i. = 5), the cells were lysed and firefly luciferase activity recorded. The antiviral activity relative to infected but non-treated cells was calculated. (b) Fourty-eight hours post infection with VSV (m.o.i. = 1), the cells were fixed with formalin containing 0.1% crystal violet, washed with water, and dried. The dye was dissolved in 70% ethanol before absorbance at 595 nm was recorded. The relative antiviral activity (%) was calculated.

To further evaluate the bioassay, samples containing type I IFN of unknown activity were tested against a commercial IFN-β standard and the results were compared with those obtained with a conventional IFN bioassay based on VSV cytotoxicity. Human type I IFN was induced in NHDF human fibroblasts following infection with VSV*M_q_ [30]. This propagation-competent virus expresses a mutant matrix protein that is unable to block the nucleocytoplasmic RNA transport of the cell. Before the samples were tested for antiviral activity, VSV*M_q_ was inactivated for 30 min with 0.1 M HCl to avoid any interference with the bioassay (see also the section on thermal and pH stability of IFN). When the VSV*ΔG(Luc) replicon bioassay was used, about 0.8 pg/ml of a commercial IFN-β standard resulted in 50% antiviral activity. In consideration of the final dilution producing 50% antiviral activity, the type I IFN concentration of two test samples was defined as 4000 pg/ml and 1440 pg/ml, respectively (Fig. 3a). When the samples were tested with the conventional bioassay (Fig. 3b), the values were in the same range (5000 and 1500 pg/ml), indicating that both assays principally agree. Nevertheless, the replicon-based bioassay proved to be more sensitive than the conventional one as lower amounts of type I IFN were sufficient to reduce the luciferase reporter activity by 50% (compare curves shown in Fig. 3a with those in Fig. 3b).

### Characterization of the species-dependent action of type I IFN

**Figure 4:**
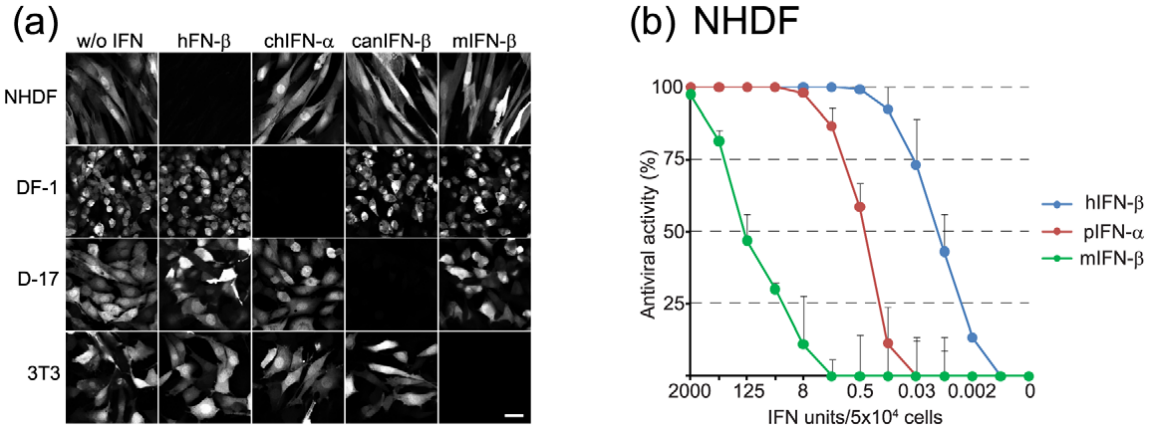
Type I IFNs act in a species-specific manner. (a) Cells derived from different species were treated for 20 h with the indicated type I IFNs (5 units/well) and subsequently infected with VSV*ΔG(Luc). Expression of GFP was monitored 6 h post infection by fluorescence microscopy. Bar, 30 µm. (b) NHDF were incubated for 20 h with serial fourfold dilutions of human, porcine and murine type I IFNs. The cells were subsequently infected with VSV*ΔG(Luc) for 5 h and firefly luciferase activity was recorded in the cell lysates.

The species-dependent action of type I IFN was analysed by incubating human, canine, murine, and chicken cells for 20 hours with 5 units of type I IFN from either the homologous species or a different one. When cells were treated with the respective homologous IFN an antiviral state was induced as indicated by the lack of GFP expression 6 hours post infection with VSV*ΔG(Luc) (Fig. 4a). In contrast, untreated cells or cells that had received type I IFN from a different species were not protected and showed GFP expression accordingly. To compare the effects of different concentrations of homologous and heterologous type I IFN on human cells, we treated NHDF for 20 hours with serial dilutions of human, porcine, and murine type I IFN prior to infection with VSV*ΔG(Luc). Firefly luciferase reporter activity in infected cell lysates indicated that porcine IFN-α is active in NHDF albeit at reduced levels compared to human IFN-β (Fig. 4b; p≤0.0049 for the 0.008 to 2.0 units range). Murine IFN-β showed an even lower activity on NHDF (ED_50_ of 500 units per 5×10^4^ NHDF cells; p≤0.0003 for the 0.008 to 500 units range). These results demonstrate that type I IFNs act in a species-dependent manner and accentuate the need for selecting appropriate cell lines to determine their activity.

### Thermal and pH stability of type I and type III IFN

**Figure 5:**
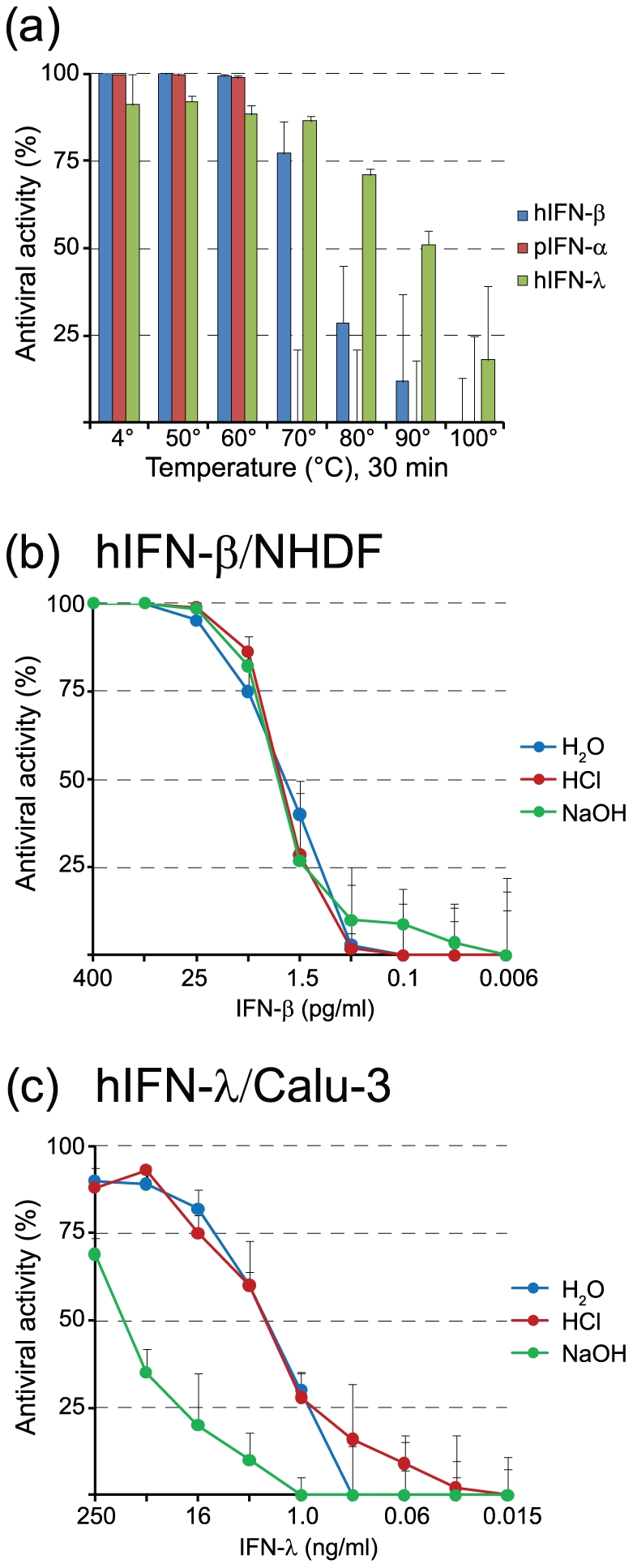
Analysis of type I IFN stability. (a) Human IFN-β and porcine IFN-α (each at 800 units/ml) were heated for 30 min at the indicated temperatures and thereafter diluted 1∶10 with medium containing 5% FBS. The heat-treated IFN preparations were incubated for 20 h with NHDF and PK-15 cells, respectively (8 units per 5×10^4^ cells). Luciferase reporter activity was determined in cell lysates 5 hours post infection with VSV*ΔG(Luc). (b) Human IFN-β and (c) human IFN-λ were incubated for 30 min at 20°C with either 0.1 M HCl, 0.1 M NaOH, or H_2_O, and then adjusted to neutral pH. NHDF and Calu-3 cells were incubated for 20 h with fourfold serial dilutions of HCl/NaOH-treated IFN-β and IFN-λ, respectively, and subsequently infected with VSV*ΔG(Luc). Firefly luciferase activity was determined in cell lysates 5 h post infection.

We often encounter the problem that the activity of type I IFN has to be determined in a sample containing live virus. As virus infection may interfere with the bioassay, it has to be inactivated before the assay is performed, preferentially without touching the activity of type I IFN. As many viruses can simply be inactivated by treatment with heat, we first analysed the thermal stability of human IFN-β using the VSV*ΔG(Luc) bioassay. The antiviral activity of human IFN-β was fully maintained when the cytokine was incubated for 30 min at 50°C (Fig. 5a). At 60°C, 99% of the activity was preserved (p = 0.021). At 70°C, the activity of human IFN-β dropped to 83% (p = 0.0017). Incubation at higher temperatures (80°C and 90°C) affected the activity more drastically, although some activity was still left at these temperatures. Only when human IFN-β was heated to 100°C for 30 min, activity was completely abolished. In contrast to human IFN-β, porcine IFN-α was completely inactivated at 70°C (p<0.0001), whereas human IFN-λ (IL-29), a type III IFN, showed even higher residual activity at 80°C and 90°C (p<0.001), suggesting that these cytokines have different physicochemical properties.

To study the sensitivity of type I and type III IFNs to conditions of extreme pH, human IFN-β and human IFN-λ were treated for 30 min with either 0.1 M HCl, 0.1 M NaOH or H_2_O, adjusted to neutral pH, and assayed on NHDF and Calu-3 cells, respectively. It turned out that the antiviral activity of human IFN-β was not significantly affected by acid (p>0.05) (Fig. 5b), confirming the previously noted acid-stability of type I IFNs [31]. IFN-λ showed similar properties as antiviral activity was maintained following treatment with 0.1 M HCl (Fig. 5c). In contrast, alkaline treatment significantly reduced the activity of IFN-λ (p<0.05 for the 250 − 1 ng/ml range), whereas IFN-β was not affected (p>0.05) (Fig. 5b). Thus, treatment with acid may be employed to inactivate virus in a test sample without affecting the activity of type I and type III IFNs. On the other hand, alkaline may be used to differentiate between type I and type III IFNs.

## Discussion

Conventional bioassays take advantage of the antiviral activity of type I IFN to measure the inhibition of virus-induced cytopathic effects in cell culture [14]. In this study, we presented an improved bioassay by employing a recombinant VSV replicon equipped with two reporter proteins. This assay proved to be advantageous over the conventional assay with respect to biosafety, sensitivity, and time requirements.

The use of cytopathic viruses in conventional type I IFN bioassays makes appropriate biosafety measures necessary to reduce the risk of unwanted virus transmission and infection. In this regard, the VSV*ΔG(Luc) replicon particles can be regarded as biosafe. Since the modified VSV genome lacks the envelope glycoprotein (G) gene, VSV*ΔG(Luc) is propagated on helper cells providing the G glycoprotein *in trans* [23]. The replicon particles produced on these cells can run a single cycle of infection but cannot produce any infectious progeny [28]. Another aspect contributing to biosafety is that the RNA replicon replicates exclusively in the cytosol and does not produce cDNA intermediates. Thus, in the case of an accidental infection any risk of recombination with or integration into host chromosomal DNA can be excluded.

Virus preparations that are used for IFN bioassays must not contain any type I IFN which may distort the results. In addition, the viruses should not induce IFN in infected reporter cells to assure that the effects measured are solely due to the exogenous IFN added. Both criteria are fulfilled for VSV*ΔG(Luc). The helper cells used to propagate the replicon particles are derived from BHK-21 cells, which are defective in the synthesis of type I IFN [32]. In addition, the VSVΔG replicon does not induce type I IFN in infected reporter cells, because the VSV matrix protein efficiently blocks the nuclear export of cellular mRNA including the IFN mRNA [30], [33].

Conventional type IFN bioassays often take 24 hours or more to be completed as they rely on the inhibition of cytopathic effects. In contrast, the bioassay presented here takes advantage of the firefly luciferase reporter, which can be detected with high sensitivity long before a cytopathic effect is apparent. Although the luciferase reporter enabled us to unambiguously quantify the IFN-mediated reduction of virus replication, we frequently observed that the standard deviations for the antiviral activities increased when the cells were treated with low amounts of type I IFN. This likely reflects the high reporter gene expression at low levels of type I IFN and the inaccuracy associated with pipetting small volumes (6 µl).

The engagement of a common cellular receptor by type I IFNs leads to activation of the JAK/STAT pathway and transcriptional induction of several genes with antiviral activity [1], [2]. It is common practice that cells are incubated with type I IFN for several hours to induce an antiviral state [15], [19]. Our findings suggest that an antiviral state can be detected much earlier. For example, treatment of NHDF with 1 unit of human IFN-β for 1 hour and subsequent infection with VSV*ΔG(Luc) for 5 hours was sufficient to suppress firefly reporter expression by 50%, even though the effective dose was further lowered with longer incubation times. While DF-1 fibroblasts responded to chicken IFN-α with dose response kinetics similar to the one observed with human IFN-β on NHDF, PK-15 cells responded to porcine type I IFN in a much slower way. Thus, compared to conventional bioassays VSV*ΔG(Luc) may allow quantification of type I IFN in a considerably shorter time provided an appropriate cell line has been selected. The selection of a suitable cell line is also important with respect to the species it is derived from, as full activity of type I IFN was only observed with cells from the corresponding species. Since VSV is able to infect a broad spectrum of mammalian and avian cells, the new bioassay may be used to determine the bioactivity of type I IFNs from many different animals.

Although type I IFNs are the predominant cytokines with antiviral activity [34], it cannot be excluded that test samples contain other cytokines that inhibit VSV replication. Indeed, using the VSV*ΔG(Luc) replicon assay, it was possible to quantify the antiviral activity of IFN-λ (Il-29), a type III IFN. This raises the important question of how to distinguish between different antiviral cytokines? Certainly, neutralizing antibodies may be employed to specify the antiviral cytokine present in the sample. In addition, the extraordinary acid-stability of type I IFNs may be used to differentiate them from acid-labile cytokines such as type II IFNs [35]. However, acid may not be used to distinguish between type I and type III IFNs as IFN-λ proved to be acid-stable as well. Since IFN-λ but not IFN-β was sensitive to 0.1 M NaOH, treatment with alkaline may be used instead. However, the conditions of treatment still have to be optimized to guarantee the complete inactivation of type III IFNs while maintaining the activity of type I IFNs.

In addition to antiviral cytokines, test samples may also contain unknown viruses that potentially interfere with the bioassay. Treatment with heat or acid may be used to inactivate these viruses without affecting the bioactivity of type I and type III IFNs. However, as heat-inactivated influenza viruses still may be able to induce type I IFN [36], virus inactivation by acid may be more convenient.

The correct and reliable determination of type I IFN biological activity is an important issue. The new bioassay presented in this study represents an attractive alternative to conventional type I IFN bioassays that work with cytotoxic live viruses. VSV*ΔG(Luc) is easily produced and handled under BSL-1 conditions. The activity of the replicon can be easily determined and standardized taking advantage of the two reporter proteins. It may be stored for prolonged time in lyophilized form without losing its activity. Finally, the proven sensitivity, rapidity, and accurateness of the assay recommends it for the determination of type I IFN from a number of mammalian and avian species. Finally, the test may be further developed to quantify the antiviral activity of other cytokines such as type II and type III IFNs.
